# Limited improvement in prostate cancer mortality-to-incidence ratios in countries with high health care expenditures

**DOI:** 10.18632/aging.103865

**Published:** 2020-11-12

**Authors:** Shao-Chuan Wang, Lung Chan, Tzuo-Yi Hsieh, Chao-Hsien Wang, Sung-Lang Chen, Wen-Wei Sung

**Affiliations:** 1 Department of Urology, Chung Shan Medical University Hospital, Taichung 40201, Taiwan; 2 School of Medicine, Chung Shan Medical University, Taichung 40201, Taiwan; 3 Institute of Medicine, Chung Shan Medical University, Taichung 40201, Taiwan

**Keywords:** prostate cancer, mortality, incidence, mortality-to-incidence ratio, expenditure

## Abstract

Prostate cancer mortality-to-incidence ratios (MIRs) are associated with the level of available healthcare. However, no data are currently available to show an association between differences in the prostate cancer MIRs and healthcare disparity. In the present study, changes in MIR over time (δMIR) were calculated as the difference between MIRs in 2018 and 2012. The significance between expenditures on healthcare and the human development index (HDI) were analyzed using Spearman's rank correlation coefficient. A total of 47 countries were studied. Countries were excluded based on inadequate data quality and missing data. The crude prostate cancer incidence rates, but not mortality rates, correlated with the HDI score and healthcare expenditure. A high HDI score and high healthcare expenditure were also significantly associated with a favorable MIR (ρ = -0.704, p < 0.001; ρ = -0.741, p < 0.001, respectively). Importantly, healthcare disparities were negatively associated with the improvement in δMIR (ρ = -0.556, p < 0.001; ρ = -0.506, p < 0.001, respectively). These findings indicate that favorable prostate cancer MIRs are associated with higher healthcare expenditures, but the trends in MIR between 2012 and 2018 correlate negatively with HDI and healthcare expenditure.

## INTRODUCTION

Prostate cancer is one of the most frequently diagnosed cancers in men and is the fifth leading cause of death worldwide. In 2018, a total of 1,276,106 new cases of prostrate cancer were diagnosed, and there were 358,989 related deaths [1]. The prognosis of patients with early stage prostate cancer is favorable compared with other malignancies; however, the high global incidence makes prostate cancer a critical health issue. Thus, understanding the global epidemiological trends for prostate cancer is a vital need.

The precise etiology of prostate cancer is unknown, but scientists generally agree that certain risk factors are closely related to its occurrence. The incidence of prostate cancer in elderly males has rapidly increased in over the past several decades [2, 3]. This may be explained by increased life expectancy and the extensive use of prostate-specific antigen (PSA) testing. The prevalence of prostate cancer also varies widely among different races. In the United States, for example, the highest incidences are among African-American men (157.6 per 100,000), while the lowest incidences are among Native Americans and Alaskans (46.9 per 100,000) and reflects the different ethnic and genetic predispositions for prostate cancer [4]. Evidence also suggests there is an association between prostate cancer and dietary and lifestyle factors. For example, smoking and obesity increase the risk of aggressive prostate cancer and mortality, whereas consumption of lycopene, cruciferous vegetables, vegetable fats, and coffee may reduce the risk of prostate cancer progression [5].

In 1970, investigators discovered that PSA expression is significantly related to the occurrence of prostate cancer, and it has since become a widely used parameter in prostate cancer screens [6, 7]. However, the low specificity of the PSA level means that indolent or potential prostate cancers are often detected. This over-detection potentially puts a large proportion of men at risk of harm due to unnecessary diagnostic procedures and treatments [8]. The European Randomized Study of Screening for Prostate Cancer (ERSPC) and the Prostate, Lung, Colorectal, and Ovarian (PLCO) cancer screening trials were supposed to help resolve the dispute over the value of PSA testing, but they ultimately arrived at opposite conclusions. The ERSPC found that PSA screening reduced prostate cancer mortality by 20%, while the PLCO found no survival benefit to PSA screening [9, 10]. Despite the divided views on the value of the PSA level as a screening parameter, the current consensus is that there is an urgent need to improve PSA testing as a screening tool to make it more effective and to reduce the overdiagnosis rate.

We were prompted to conduct the present study because of our interest in the impact of changes to screening policy and advances in therapeutic strategy over the past few years. The mortality-to-incidence ratio (MIR) has been identified as an innovative parameter that has been used as a valid proxy for the 5-year relative survival rate in many types of cancer [11], though a recent study came to an opposite conclusion regarding the prognostic role of MIR [12].

We addressed this controversy by analyzing the trends in MIR; specifically, the changes in MIR (δMIR) between 2012 and 2018, which enabled observation of the changes in prostate cancer prognosis in different countries. The aim of the present study was to elucidate the association between various factors, including the human development index (HDI), current healthcare expenditure (CHE), incidence and mortality, and the MIR for prostate cancer. The results provide a more comprehensive understanding of the association between the prostate cancer MIR and the level of socioeconomic development in different countries.

## RESULTS

### Regional differences in prostate cancer incidence and mortality

The incidence and mortality number, age standardized rate (ASR), crude rate (CR), and MIR for prostate cancer are summarized in Table 1. When ranking regions based on the HDI, very high-HDI regions had the highest CR for prostate cancer incidence and mortality (117.0 and 23.1, respectively), but the lowest MIR (0.20). Medium-HDI regions had the lowest CR for incidence and mortality (6.7 and 3.5, respectively), while the MIR was highest in the low-HDI regions (0.58). When arranging these regions by continent, three regions had a CR for prostate cancer incidence greater than 100, North America (130.0), Europe (125.1) and Oceania (113.8). The CR for mortality in the latter two regions was also greater than 20.0, though not for North America (18.1). Regions with a lower CR for prostate cancer incidence and mortality included Africa and Asia (Africa: 6.7 and 3.5, respectively; Asia: 6.7 and 3.5, respectively). Africa had the highest MIR (0.52), while North America had the lowest MIR (0.14).

**Table 1:** Summary of the regional prostate cancer incidences, mortality rates, and mortality-to-incidence ratios.

**Region**	**Incidence**			**Mortality**			**MIR**
	**Number**	**ASR^1^**	**CR^1^**	**Number**	**ASR^1^**	**CR^1^**	
HDI							
Very High HDI	802294	61.1	117.0	158335	8.9	23.1	0.20
High HDI	225363	19.6	26.1	68309	7.0	9.7	0.37
Medium HDI	68381	8.6	6.7	31770	4.5	3.5	0.52
Low HDI	53890	26.1	10.5	31129	15.9	6.1	0.58
Continent							
Africa	80971	26.6	12.6	42298	14.6	6.6	0.52
Asia	297215	11.5	12.8	118427	4.5	5.1	0.40
Europe	449761	62.1	125.1	107315	11.3	29.8	0.24
Latin America and Caribbean	190385	56.4	59.1	53798	14.2	16.7	0.28
Northern America	234278	73.7	130.0	32686	7.7	18.1	0.14
Oceania	23496	79.1	113.8	4465	10.7	21.6	0.19

ASR, age-standardized rate; CR, crude rate; HDI, human development index; MIR, mortality-to-incidence ratio

^1^per 100,000

### Incidences, mortalities, and MIRs for prostate cancer as well as HDIs in different countries

Supplementary Table 1 summarizes the HDI, CHE, incidence, mortality, and MIR for prostate cancer in selected countries. The five countries with the highest CR for incidence were Ireland (206.8), Sweden (206.8), Estonia (197.6), Norway (195.1) and France (194.8). The five countries with the highest CR for mortality were Estonia (40.5), Latvia (39.1), Trinidad and Tobago (33.9), Lithuania (33.5) and Denmark (30.2). Two countries have MIRs over 0.40, Ukraine (0.44) and Thailand (0.42), while three have MIRs below 0.10, Luxembourg (0.09), Ireland (0.08) and France (0.08). Comparison of the 2012-2018 δMIRs revealed that the five countries with the highest δMIRs were the Philippines (0.18), Argentina (0.13), Thailand (0.12), Belarus (0.12) and Costa Rica (0.12). The countries with the lowest δMIRs were Canada (0.02), Finland (0.01), Israel (0.00), Latvia (-0.01) and Germany (-0.01).

### Association between CRs for incidence and mortality, HDIs, per capita CHEs, CHEs/GDP, MIRs and δ-MIRs in different countries

**Figure 1:**
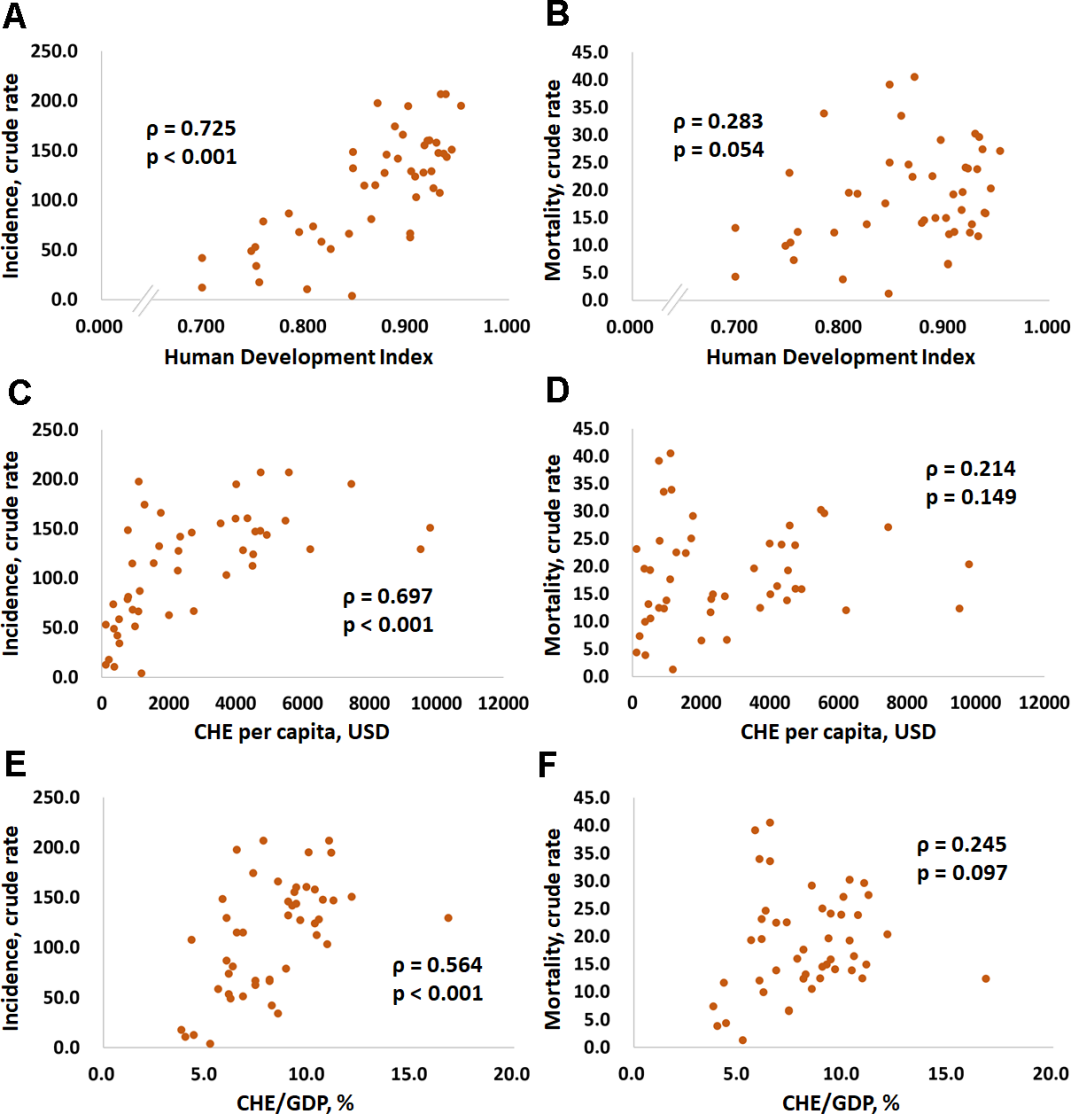
****Association between the human development index, current health expenditures, and human development index and the crude rates of incidence (**A**, **C**, and **E**) and mortality (**B**, **D**, and **F**) in prostate cancer.

**Figure 2:**
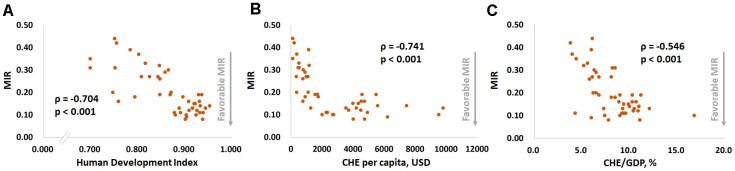
****The (**A**) human development index, (**B**) current per capita health expenditure, and (**C**) current health expenditure as a percentage of the gross domestic product are significantly associated with the mortality-to-incidence ratio (MIR) in prostate cancer.

**Figure 3:**
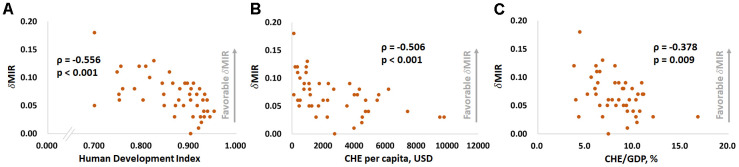
****The (**A**) human development index, (**B**) current per capita health expenditure, and (**C**) current health expenditure as a percentage of gross domestic product are significantly associated with the change in the prostate cancer mortality-to-incidence ratio (δMIR) from 2012 to 2018.

Figures 1 and 2 show the correlations between the CRs for prostate cancer incidence and mortality and the HDI, per capita CHE, CHE/GDP and MIR in selected countries. Figures 1A, 1C, and 1E show that the CRs for incidence have a significant positive correlation with the HDI, per capita CHE, and CHE/GDP (ρ = 0.725, p < 0.001, Figure 1A; ρ = 0.697, p < 0.001, Figure 1C; ρ = 0.564, p < 0.001, Figure 1E). However, Figures 1B, 1D, and 1F show that the CRs for mortality do not significantly correlate with the HDI, per capita CHE, or CHE/GDP (ρ = 0.283, p = 0.054, Figure 1B; ρ = 0.214, p = 0.149, Figure 1D; ρ = 0.245, p = 0.097, Figure 1F). Figures 2A, 2B, and 2C show that a high HDI, per capita CHE, and CHE/GDP all significantly associate with favorable MIRs (ρ = -0.704, p < 0.001, Figure 2A; ρ = -0.741, p < 0.001, Figure 2B; ρ = -0.546, p < 0.001, Figure 2C, respectively). Figures 3A–3C show negative trends between δMIR and HDI, per capita CHE, and CHE/GDP. Countries with low HDIs, per capita CHEs, and CHEs/GDP tend to have favorable δMIRs (ρ = -0.556, p < 0.001, Figure 3A; ρ = -0.506, p < 0.001, Figure 3B; ρ = -0.378, p = 0.009, Figure 3C, respectively).

## DISCUSSION

The results presented here confirm the significant association between prostate cancer crude incidence and mortality and MIR and the level of socioeconomic development. The HDI, per capita CHE, and CHE/GDP are all significantly positively related to prostate cancer crude incidence and mortality (Figure 1). The HDI, per capita CHE, and CHE/GDP also correlate with a favorable prostate cancer MIR (Figure 2). These correlations indicate that better developed countries tend to have higher prostate cancer incidences but lower MIRs.

The high prevalence in more developed regions may be evidence that diet, lifestyle, and environmental influences are risk factors for prostate cancer. For example, people who live in more developed regions are more easily exposed to carcinogens that promote prostate cancer, such as cigarettes and processed red meats [5, 13]. Excessive calcium and choline intake from dietary sources also increase the risk of prostate cancer [14, 15]. Conversely, we believe that the early detection and appropriate treatment of prostate cancer may explain the trend toward lower MIRs in more developed regions. As with cigarettes and processed red meat, the reason is again greater access; people living in more developed regions have greater access to higher levels of healthcare. For example, novel screening tools and treatments such as androgen deprivation therapy and bone-supportive agents are more accessible in highly developed areas [16].

In this study, δMIR was used to evaluate the trends in MIR over time. A higher δMIR means that countries have made more progress from 2012 to 2018. As mentioned, countries with better socioeconomic conditions often tend to have lower MIRs (Figure 2). Notably, however, the δMIR is negatively correlated with the socioeconomic development level (Figure 3), which indicates that the better-developed countries show less progress with prostate cancer prognosis during the aforementioned period. The reason for this may be that by 2012 the prognosis of prostate cancer patients was already relatively good in more developed countries, so progress in the past few years would be only incremental, even with more precise treatments [2, 3]. It may also indicate that when evaluating the effectiveness of treatment, prostate cancer prognosis based on the survival rate should not be the only indicator. Other factors, such as the quality of life and cancer-related complications, also require consideration [17, 18].

Our study has some limitations. We analyzed data from a total of 47 countries, but we excluded countries for which the data quality was poor or data assessments were unavailable. This incomplete data collection may reduce the generalizability of our results. The data on prostate cancer incidence and mortality estimates were also obtained from the GLOBOCAN 2012 and 2018 databases and are subject to accuracy limitations related to large inequalities in the access to high-quality local data in transitioning countries [1]. In addition, there are no time series data for this period, and the prognostic utility of MIR cannot replace the data from cohort observations [12]. Further investigation with cohort surveys and detailed clinical-pathological and therapeutic information will be necessary.

Despite these limitations, the present study demonstrates that countries with a higher level of socioeconomic development tend to have a better prognosis for prostate cancer. The δMIRs, as longitudinal data, enable us to observe the trend in prostate cancer prognosis and helps us to monitor improvements in prostate cancer care in different countries. Our study also confirms that the MIR has a role when assessing the availability of healthcare in different countries and emphasizes the need to reduce healthcare disparities.

## MATERIALS AND METHODS

Cancer epidemiological data were obtained from the GLOBOCAN project (http://gco.iarc.fr/), a public access database that provides contemporary estimates of cancer epidemiology for 185 countries or territories of the world in 2018. The GLOBOCAN project is maintained by the International Agency for Research on Cancer, World Health Organization. The exclusion criteria for country selection were based on the data quality report in GLOBOCAN (N=123) and missing data (N=7). Outliers of the 2018 MIR (N=1) and δMIR (N=9) were also excluded. A total of 47 countries were included in the final analysis.

The HDI was obtained from the United Nations Development Programme, Human Development Report Office (http://hdr.undp.org/en). The data on health expenditures, including the per capita CHE and CHE/GDP (ratio of CHE to the % of GDP, gross domestic product), were obtained from the World Health Statistics database (https://www.who.int/gho/publications/world_health_statistics/en/). The MIR was defined as the ratio of the CR of mortality to the CR of incidence, as previously described [19–22]. The δMIR was defined as the difference between the MIR in 2012 and 2018 (δMIR = MIR [in 2012] - MIR [in 2018]). Associations between the MIR, δMIR, and other factors among various countries were estimated using Spearman's rank correlation coefficient calculated using SPSS statistical software version 15.0 (SPSS, Inc., Chicago, IL). Values of *P* < 0.05 were considered statistically significant. Scatterplots were generated with Microsoft Excel.

## Supplementary Material

Supplementary Table 1
