# Supplementary material for: Limited improvement in prostate cancer mortality-to-incidence ratios in countries with high health care expenditures
Source: Aging (Albany NY). 2020 Nov 12;12(21):21308–15. doi: 10.18632/aging.103865 (PMC7695365; doi:10.18632/aging.103865)
Supplement: Supplementary Table 1 [file aging-12-103865-s001..docx]

**Supplementary Table 1. Summary of the human development index, current health expenditures, cancer incidences, cancer mortality, and mortality-to-incidence ratios for prostate cancer in selected countries.**

|  | Human Development Index | |  | Current Health Expenditure | |  | Incidence | | |  | Mortality | | |  | Mortality-to-incidence Ratio | | |
| --- | --- | --- | --- | --- | --- | --- | --- | --- | --- | --- | --- | --- | --- | --- | --- | --- | --- |
| Country | Score | Rank |  | Per Capita | % of GDP |  | ASR^1^ | CR^1^ | Cumulative Risk |  | ASR^1^ | CR^1^ | Cumulative Risk |  | 2012 | 2018 | 𝛿MIR |
| Argentina | 0.825 | 47 |  | 998 | 6.8 |  | 41.0 | 51.0 | 5.6 |  | 9.5 | 13.8 | 1.0 |  | 0.40 | 0.27 | 0.13 |
| Australia | 0.939 | 3 |  | 4934 | 9.4 |  | 83.9 | 143.5 | 10.8 |  | 6.6 | 15.8 | 0.7 |  | 0.15 | 0.11 | 0.04 |
| Austria | 0.908 | 20 |  | 4536 | 10.3 |  | 59.4 | 123.8 | 7.7 |  | 6.6 | 19.2 | 0.7 |  | 0.19 | 0.16 | 0.03 |
| Bahrain | 0.846 | 43 |  | 1190 | 5.2 |  | 9.7 | 3.7 | 1.2 |  | 3.2 | 1.2 | 0.3 |  | 0.39 | 0.32 | 0.07 |
| Belarus | 0.808 | 53 |  | 352 | 6.1 |  | 47.2 | 73.5 | 6.3 |  | 12.2 | 19.5 | 1.6 |  | 0.39 | 0.27 | 0.12 |
| Belgium | 0.916 | 17 |  | 4228 | 10.5 |  | 64.0 | 127.9 | 8.5 |  | 6.0 | 16.4 | 0.6 |  | 0.20 | 0.13 | 0.07 |
| Brazil | 0.759 | 79 |  | 780 | 8.9 |  | 70.9 | 78.6 | 9.3 |  | 10.2 | 12.4 | 1.1 |  | 0.24 | 0.16 | 0.08 |
| Canada | 0.926 | 12 |  | 4508 | 10.4 |  | 56.5 | 112.2 | 7.5 |  | 5.4 | 13.8 | 0.6 |  | 0.14 | 0.12 | 0.02 |
| Chile | 0.843 | 44 |  | 1102 | 8.1 |  | 47.0 | 66.1 | 6.1 |  | 11.2 | 17.6 | 1.1 |  | 0.36 | 0.27 | 0.09 |
| Colombia | 0.747 | 90 |  | 374 | 6.2 |  | 46.4 | 48.7 | 6.1 |  | 9.0 | 9.9 | 0.9 |  | 0.31 | 0.20 | 0.11 |
| Costa Rica | 0.794 | 63 |  | 929 | 8.1 |  | 53.8 | 67.8 | 7.0 |  | 8.3 | 12.3 | 0.8 |  | 0.30 | 0.18 | 0.12 |
| Cyprus | 0.869 | 32 |  | 1563 | 6.8 |  | 67.7 | 114.9 | 8.7 |  | 10.2 | 22.4 | 0.9 |  | 0.22 | 0.19 | 0.03 |
| Czechia | 0.888 | 27 |  | 1284 | 7.3 |  | 86.6 | 174.2 | 11.5 |  | 9.2 | 22.5 | 1.0 |  | 0.18 | 0.13 | 0.05 |
| Denmark | 0.929 | 11 |  | 5497 | 10.3 |  | 73.8 | 157.8 | 9.8 |  | 10.2 | 30.2 | 1.0 |  | 0.25 | 0.19 | 0.06 |
| Ecuador | 0.752 | 86 |  | 530 | 8.5 |  | 34.2 | 33.8 | 4.3 |  | 9.4 | 10.5 | 0.9 |  | 0.37 | 0.31 | 0.06 |
| Estonia | 0.871 | 30 |  | 1112 | 6.5 |  | 106.9 | 197.6 | 13.9 |  | 17.5 | 40.5 | 1.9 |  | 0.25 | 0.20 | 0.05 |
| Finland | 0.920 | 15 |  | 4005 | 9.4 |  | 68.1 | 159.9 | 8.8 |  | 8.0 | 24.1 | 0.8 |  | 0.16 | 0.15 | 0.01 |
| France | 0.901 | 24 |  | 4026 | 11.1 |  | 96.8 | 194.8 | 12.6 |  | 5.2 | 14.9 | 0.5 |  | 0.15 | 0.08 | 0.07 |
| Germany | 0.936 | 5 |  | 4592 | 11.2 |  | 60.7 | 146.8 | 8.1 |  | 8.2 | 27.4 | 0.9 |  | 0.18 | 0.19 | -0.01 |
| Ireland | 0.938 | 4 |  | 4757 | 7.8 |  | 131.2 | 206.8 | 16.5 |  | 7.3 | 15.9 | 0.7 |  | 0.14 | 0.08 | 0.06 |
| Israel | 0.903 | 22 |  | 2756 | 7.4 |  | 50.8 | 66.5 | 7.0 |  | 4.1 | 6.6 | 0.4 |  | 0.10 | 0.10 | 0.00 |
| Italy | 0.880 | 28 |  | 2700 | 9.0 |  | 59.8 | 145.9 | 8.1 |  | 4.0 | 14.5 | 0.4 |  | 0.18 | 0.10 | 0.08 |
| Japan | 0.909 | 19 |  | 3733 | 10.9 |  | 33.1 | 103.1 | 4.4 |  | 3.0 | 12.4 | 0.3 |  | 0.21 | 0.12 | 0.09 |
| Latvia | 0.847 | 41 |  | 784 | 5.8 |  | 76.8 | 148.4 | 10.1 |  | 16.8 | 39.1 | 2.0 |  | 0.25 | 0.26 | -0.01 |
| Lithuania | 0.858 | 35 |  | 923 | 6.5 |  | 68.9 | 114.6 | 8.3 |  | 14.8 | 33.5 | 1.7 |  | 0.40 | 0.29 | 0.11 |
| Luxembourg | 0.904 | 21 |  | 6236 | 6.0 |  | 76.8 | 129.2 | 10.6 |  | 5.7 | 12.0 | 0.7 |  | 0.17 | 0.09 | 0.08 |
| Malaysia | 0.802 | 57 |  | 386 | 4.0 |  | 11.4 | 10.4 | 1.6 |  | 4.0 | 3.8 | 0.4 |  | 0.43 | 0.37 | 0.06 |
| Malta | 0.878 | 29 |  | 2304 | 9.6 |  | 53.8 | 127.3 | 7.2 |  | 5.2 | 14.0 | 0.6 |  | 0.17 | 0.11 | 0.06 |
| Netherlands | 0.931 | 10 |  | 4746 | 10.7 |  | 67.9 | 147.5 | 9.2 |  | 8.4 | 23.8 | 0.9 |  | 0.20 | 0.16 | 0.04 |
| New Zealand | 0.917 | 16 |  | 3554 | 9.3 |  | 87.6 | 155.1 | 11.3 |  | 8.5 | 19.6 | 0.9 |  | 0.18 | 0.13 | 0.05 |
| Norway | 0.953 | 1 |  | 7464 | 10.0 |  | 103.6 | 195.1 | 13.4 |  | 10.9 | 27.1 | 1.1 |  | 0.18 | 0.14 | 0.04 |
| Philippines | 0.699 | 113 |  | 127 | 4.4 |  | 18.5 | 12.2 | 2.2 |  | 6.4 | 4.3 | 0.5 |  | 0.53 | 0.35 | 0.18 |
| Poland | 0.865 | 33 |  | 797 | 6.3 |  | 42.4 | 80.9 | 5.9 |  | 11.6 | 24.6 | 1.3 |  | 0.39 | 0.30 | 0.09 |
| Portugal | 0.847 | 41 |  | 1722 | 9.0 |  | 58.3 | 132.1 | 7.9 |  | 7.4 | 25.0 | 0.7 |  | 0.24 | 0.19 | 0.05 |
| Russian Federation | 0.816 | 49 |  | 524 | 5.6 |  | 38.2 | 58.2 | 5.3 |  | 12.1 | 19.3 | 1.6 |  | 0.43 | 0.33 | 0.10 |
| Singapore | 0.932 | 9 |  | 2280 | 4.3 |  | 57.8 | 107.4 | 7.2 |  | 5.9 | 11.6 | 0.6 |  | 0.14 | 0.11 | 0.03 |
| Slovenia | 0.896 | 25 |  | 1772 | 8.5 |  | 77.4 | 165.9 | 10.3 |  | 10.4 | 29.1 | 1.0 |  | 0.27 | 0.18 | 0.09 |
| South Africa | 0.699 | 113 |  | 471 | 8.2 |  | 61.0 | 41.9 | 7.5 |  | 20.3 | 13.1 | 2.2 |  | 0.36 | 0.31 | 0.05 |
| South Korea | 0.903 | 22 |  | 2013 | 7.4 |  | 34.4 | 62.4 | 4.7 |  | 3.1 | 6.5 | 0.3 |  | 0.16 | 0.10 | 0.06 |
| Spain | 0.891 | 26 |  | 2354 | 9.2 |  | 73.2 | 141.9 | 10.1 |  | 5.2 | 14.9 | 0.5 |  | 0.20 | 0.11 | 0.09 |
| Sweden | 0.933 | 7 |  | 5600 | 11.0 |  | 101.2 | 206.8 | 13.2 |  | 9.6 | 29.6 | 0.9 |  | 0.21 | 0.14 | 0.07 |
| Switzerland | 0.944 | 2 |  | 9818 | 12.1 |  | 74.4 | 150.7 | 9.8 |  | 7.1 | 20.3 | 0.7 |  | 0.16 | 0.13 | 0.03 |
| Thailand | 0.755 | 83 |  | 217 | 3.8 |  | 11.3 | 17.5 | 1.5 |  | 4.5 | 7.3 | 0.6 |  | 0.54 | 0.42 | 0.12 |
| Trinidad and Tobago | 0.784 | 69 |  | 1146 | 6.0 |  | 65.0 | 86.7 | 8.3 |  | 24.1 | 33.9 | 2.4 |  | 0.47 | 0.39 | 0.08 |
| Ukraine | 0.751 | 88 |  | 125 | 6.1 |  | 31.0 | 53.0 | 4.3 |  | 12.9 | 23.1 | 1.8 |  | 0.51 | 0.44 | 0.07 |
| United Kingdom | 0.922 | 14 |  | 4356 | 9.9 |  | 77.3 | 160.2 | 10.1 |  | 8.5 | 23.9 | 0.9 |  | 0.23 | 0.15 | 0.08 |
| United States of America | 0.924 | 13 |  | 9536 | 16.8 |  | 74.6 | 129.2 | 9.8 |  | 5.8 | 12.3 | 0.7 |  | 0.13 | 0.10 | 0.03 |

ASR, age-standardized rate; CR, crude rate; GDP, gross domestic product; MIR, mortality-to-incidence ratio

^1^per 100,000
